# Translational Value of Skilled Reaching Assessment in Clinical and Preclinical Studies on Motor Recovery After Stroke

**DOI:** 10.1177/15459683211005022

**Published:** 2021-04-07

**Authors:** Eline C. C. van Lieshout, Julia Boonzaier, Adam J. Pel, Caroline L. van Heijningen, Jord J. Vink, Johanna M. A. Visser-Meily, Geralda A. F. van Tilborg, Rick M. Dijkhuizen

**Affiliations:** 1 University Medical Center Utrecht, Utrecht University, Utrecht, Netherlands; 2 De Hoogstraat Rehabilition Utrecht, Utrecht, Netherlands

**Keywords:** skilled reaching, stroke, upper limb, recovery, behavioral measures, clinical outcome measures

## Abstract

**Background:**

Assessment of skilled reaching enables extensive analysis of upper limb function in clinical and preclinical studies on poststroke outcome. However, translational research if often limited by lack of correspondence between tests of human and rodent motor function.

**Objectives:**

To determine (1) the translational value of skilled reaching performance for preclinical research by comparing the behavioral recovery profiles of skilled reaching characteristics between humans and rats recovering from stroke and (2) the relationship between skilled reaching performance and commonly used clinical outcome measures after stroke.

**Methods:**

Twelve patients with ischemic or hemorrhagic stroke and 17 rats with photothrombotic stroke underwent an equivalent skilled reaching test at different time points, representing early to late subacute stages poststroke. Success scores and a movement element rating scale were used to measure the skilled reaching performance. The Fugl-Meyer Upper Extremity (FM-UE) assessment and the Action Research Arm Test (ARAT) were used as clinical outcome measures.

**Results:**

Both species had muscle flaccidity at the early subacute stage after stroke and showed motor recovery following a proximal-distal principle toward the early subacute stage, albeit for rats within a shorter time course. Human skilled reaching scores and FM-UE and ARAT scores in the first 3 months poststroke were significantly correlated (*P* < .05).

**Conclusions:**

Our study demonstrates that poststroke changes in skilled reaching performance are highly similar between rats and humans and correspond with standard clinical outcome measures. Skilled reaching testing therefore offers an effective and highly translational means for assessment of motor recovery in experimental and clinical stroke settings.

## Introduction

Upper limb impairment, such as a hemiparesis of the contralateral limb, is diagnosed in about 75% of the stroke patient population.^1,2^ Upper limb impairment limits functional independence, participation in social roles, and a return to work.^3^ Impairments in skilled use of the hands are cardinal features of poststroke motor dysfunction,^4^ and compensatory movement patterns, for example, excessive trunk displacement and increased reliance on the nonparetic hand, are common responses to hand function loss.^5^

Compensation is often mistaken for recovery, since some compensatory movement patterns are subtle enough to be undetected in clinical outcome measures that focus little on qualitative aspects of movement.^6^ In the process of poststroke motor recovery, early adoption of compensation strategies may lead to learned disuse or training-induced misuse of the impaired limb, which in the long term can limit a patient’s rehabilitation.^7^ In addition, when recovery is evaluated without taking compensation into account, this could distort understanding of the contribution of neural plasticity to poststroke recovery. This is particularly relevant in basic neuroscience studies and translational research on spontaneous recovery or restorative treatments,^6,7^ which often make use of animal models, mostly involving rodents. However, translational research is often limited by lack of correspondence between tests of human and rodent motor function.^8,9^ Skilled reaching assessment has been proposed as one of the most potent translational behavioral tests for studying poststroke recovery in rodents.^9^ The typical task requires that a subject reaches for and subsequently grasps a small food item with a single hand/paw, and subsequently brings it to the mouth for eating.^10^ Skilled reaching (conventional term for reach-to-eat) movement patterns show significant homologies between rodents and humans,^11-13^ which offers valuable opportunities for translational research.

Guidelines to enhance the alignment of preclinical and clinical stroke recovery research pipeline have recently been published by the Stroke Recovery and Rehabilitation Roundtable consortium.^8^ Behavioral outcome measures have received special consideration, and it has been recommended that clinically relevant deficits, such as skilled reaching, should be the main focus of preclinical behavioral testing.^14^ The time course of recovery is more rapid in rodents than in humans,^14,15^ and it remains to be determined to what extent behavioral recovery profiles in rodent stroke models are representative for functional recovery patterns in stroke patients. To our knowledge, the development of skilled reaching performance over time after stroke has not yet been directly compared between rats and humans. Therefore, the aim of our study was to determine the degree of correspondence of temporal changes in several skilled reaching characteristics in rats and humans between early and late subacute stages poststroke. To further evaluate the translational value of skilled reaching, our second aim was to determine the relationship between skilled reaching performance and commonly used clinical outcome measures (ie, Fugl-Meyer Upper Extremity assessment [FM-UE] and Action Research Arm Test [ARAT] scores) in subacute stroke patients.

## Methods

### Stroke Patients

Human data for this study were collected from the B-STARS trial, which assesses the effect of repetitive transcranial magnetic brain stimulation (rTMS) on upper limb recovery after stroke.^16^ Patients were included from whom complete skilled reaching, FM-UE, and ARAT data were available for the first 3 months after stroke. The B-STARS trial, which was ongoing at the time of submission of the current article, is a stratified, randomized controlled trial consisting of a 2-week rTMS or sham-stimulation treatment starting at 2 to 3 weeks poststroke. Twelve patients from both treatment groups were included, and investigators were blinded for group assignments. Patients with completed skilled reaching assessments between November 2018 and November 2019 were included. At 7 time points, multiple performance assays and functional tasks have been conducted to monitor upper limb recovery, among which were a skilled reaching task, FM-UE assessment, and the ARAT (details described below). Outcomes from assessment at 2 to 3 weeks (before intervention), 4 to 5 weeks (28-35 days; no FM-UE assessment), 6 weeks (40-44 days), 9 weeks (59-67 days), and 11 to 15 weeks (76-104 days) poststroke were used for the current study. Eleven out of 12 patients underwent MRI sessions at 5 to 6 weeks, 11 to 15 weeks, 22 to 26 weeks, and/or 46 to 50 weeks (see Supplementary File A, available online). All patients were inpatients of a rehabilitation facility in the Netherlands. The inpatient rehabilitation program consisted of a multidisciplinary approach to reach complex (physical and cognitive) rehabilitation goals. Full details of the B-STARS trial have been reported elsewhere.^16^

The B-STARS protocol has been approved by the Medical Ethics Committee of the University Medical Center Utrecht and the participating rehabilitation center. The trial is registered in the Dutch Trial Register (Trial NL5952). All patients gave (written) informed consent to participate in the trial.

### Rat Stroke Model

Animal data were collected from a randomized controlled preclinical trial, which was ongoing at the time of submission of the current article, to assess the effects of rTMS (vs sham stimulation) on forelimb recovery after unilateral photothrombotic stroke in the forelimb region of the sensorimotor cortex. Seventeen rats from both treatment groups were included, and investigators were blinded for group assignments. Details on the photothrombotic stroke induction and rTMS protocols can be found in Supplementary File A (available online). Seventeen male Sprague Dawley rats (Charles River Laboratories; 326 ± 26 g, 10-11 weeks old at the time of stroke induction) underwent skilled reaching tests (details described below) at days 0 (prestroke), 3, 9, 16, and 23 poststroke. Animals underwent MRI, which included anatomical MRI for lesion detection (see Supplementary File A, available online) at 2, 17, and 24 days poststroke. All experiments were approved by the Animal Ethics Committee of the University Medical Center Utrecht, the Netherlands, and were conducted in agreement with Dutch laws (“Wet op de Dierproeven,” 1996) and European regulations (Guideline 86/609/EEC).

The animals were housed under a regular 12-hour light/dark cycle and at constant temperature (24 °C, 45% to 65% humidity). Prior to the stroke induction surgery, the rats underwent skilled reaching training and were housed 2 or 3 in per standard cage (30 × 40 × 20 cm^3^). During skilled reaching training (described in Supplementary File A, available online), the animals were food-restricted to reach 90% of their initial body weight by receiving 26 to 30 g of food per cage (daily), with water freely available.

Three days after stroke induction, the animals were moved to an enriched environment, as a standard procedure, representing a clinical rehabilitation setting.^17^ While the animals were housed in groups of 4 to 5 in the enriched environment, they were only food-deprived during the dark cycle before a skilled reaching test.

### Skilled Reaching Task: Humans

**Figure 1.**
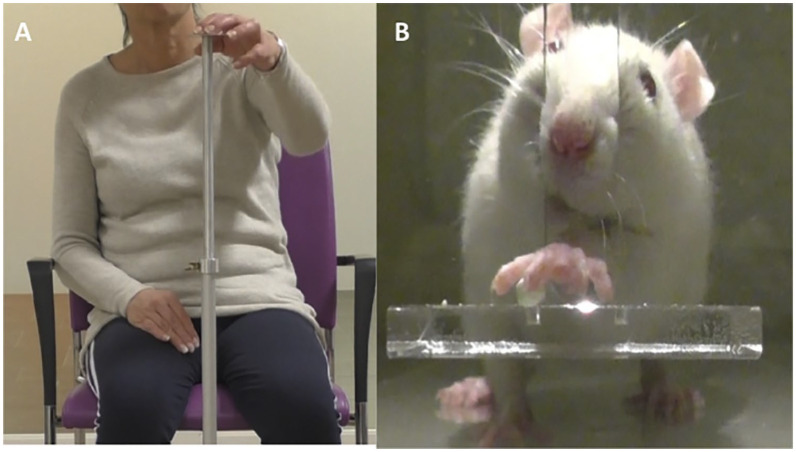
Setup of the skilled reaching task for humans (A) and rats (B) (single frontal image from video recording).

Stroke patients were seated in a chair, with their feet flat on the ground and hands palm down on their thighs with the fingers extended. A small food item (Kellogg’s Honey Pops Loops) was placed on a pedestal positioned in front of the patient, adjusted to their trunk height and arm length at full extension (10 cm beneath the outstretched palm; see Figure 1A). The patient was asked, in a standardized way by the researcher, to reach for the food item, grasp it, and place it into the mouth for eating. The skilled reaching test was performed once for the nonparetic side and at least once (out of maximally 5 attempts) for the paretic side at each time point.

### Skilled Reaching Task: Rats

Rats were placed in a rectangular skilled reaching box made of transparent Plexiglas (Figure 1B). The animals were trained to grasp (at least 20) sugar pellets through a vertical slot (1 cm wide, extended 3 cm above the floor) in the front wall of the box. On the outside of the wall, in front of the slot, mounted 3 cm above the floor, was a shelf with an indentation allowing pellet placement slightly lateral to the opening of the slot. This off-center positioning of the pellet forced rats to use their dominant/preferred forelimb (determined during the training phase) to obtain the target. For the study, the rats executed 20 trials. However, in some cases (21% of all sessions) less trials were executed due to impairment or motivation issues. Details on the skilled reaching (schedule) and food restriction protocols can be found in Supplementary File A (available online).

### Video Recording

High-speed video recording from the frontal perspective was used for humans (Panasonic HC-V770) and rats (Panasonic HC-V520) with a shutter speed set at 1/1000 frames per second, to produce a blur-free image for frame-by-frame playback (see Figure 1). For recordings of skilled reaching in rats, 2 specific light sources were used.

The recordings were analyzed using VLC media player (VideoLAN). Analyses of the human and rodent data were performed independently by a clinical (AJP) and preclinical researcher (CLvH), respectively.

### Skilled Reaching Performance

Reaching behavior was analyzed (1) from the end-point measure of success and (2) with a movement element scoring system. A reach was defined as a success when the food item was grasped, transported by the hand or paw, and placed into the mouth.

Success percentage was expressed as the number of subjects (patients or rats) that could execute a successful, fully completed reach (ie, food item was grasped, transported by the hand or paw, and placed into the mouth), divided by the total number of subjects, for each individual time point. This adapted measure of successful reaching was used for comparison across species. Stroke patients executed the reaching task up to maximally 5 times, to limit the time and the impact of fatigue and frustration associated with repetitive task executions and recurrent failures, respectively. For rats, the conventional measure of success rate, that is, the percentage of successfully obtained pellets with respect to the number of trials, was also calculated.^18^

Skilled reaching performance was scored using a biometric rating scale, based on a conceptual framework derived from the Eshkol-Wachmann Movement Notation (EWMN).^19^ The EWMN describes the position of the individual limbs, the trunk, the snout (rat), and the head in relation to the body or the food (pellet), with the body treated as a system of axes (ie, limb segments, trunk axis, and snout axis [rat]). For stroke patients, the best attempt (fully or furthest completed) with the paretic limb was selected for movement analysis with the Eshkol-Wachmann Movement Notation-Derived Reaching Scale (EW-DRS). This scale is divided into 7 elements: orient, lift, advance, pronate, grasp, supinate, and release. Each element is described with regard to its proper execution, for example, “initial hand lift is due to flexion of the elbow” and “trunk leans to the side opposite to the reach as hand approaches the target.”^4,9^ The elements were further divided into 2 or more subelements that are rated on a 3-point ordinal scale, from 0 (movement was normal) to 0.5 (movement was present but abnormal or incomplete) to 1 (movement was absent). Elements that could not be executed were scored as no movement (ie, score of 1). The overall score was the sum of all subelement scores (possible range: 0-21), with lower scores representing better performance. Sum scores were converted to a 0 to 1 scale by dividing by the maximum score, to allow direct comparison between the movement elements and against corresponding rat data.

For each rat, the 3 best reaches (fully or furthest completed) for each session were scored, averaged, and selected for further movement analysis with an EW-DRS adapted to Sprague-Dawley rats.^18^ Eleven elements of the reaching behavior were scored, with a similar scoring system as described for the humans. The total overall score was the sum of the element scores (possible range: 0-11). The sum score was converted to a 0 to 1 scale, as described above for the human data.

A full description of the skilled reaching performance scoring in humans and rats is provided in Supplementary File B (available online).

### Fugl-Meyer Upper Extremity Assessment

Stroke patients underwent the FM-UE assessment, which is a standard motor performance-based test consisting of 33 tasks performed with the affected upper limb.^20,21^ The FM-UE assessment focuses on upper limb motor impairment with regard to synergistic motor control. Performance on each task was scored on a 3-point scale, with higher ratings representing better performance (possible range = 0-66 points).^22^

### Action Research Arm Test

Stroke patients underwent the ARAT, which is a performance test that assesses the ability to perform gross movements and the ability to grasp, move, and release objects differing in size, weight, and shape.^23^ The test consists of 19 items, rated on 4-point ordinal scale (0-3), with a maximum score of 57 (best performance).

### Statistical Analysis

Generalized linear mixed models for human data were used to compare skilled reaching performance on the movement subelements at each time point poststroke in comparison to the first measurement. This data were treated as interval data as defined by Field and Hole,^24^ and therefore an ordinal logistic regression approach was used. In the analyses we included a random intercept, and time as a fixed effect. Linear mixed models for human and rat data were used to compare reaching performance on the movement elements (rat) and sum scores (human and rat) at each time point poststroke.

Linear mixed models were also used to assess temporal changes in patients’ relative sum scores (normalized to a percentage of the maximum score of that particular test) for the skilled reaching, ARAT, and FM-UE assessments, as well as their mutual relationships. For comparison with the ARAT and FM-UE scoring system, skilled reaching movement scores were reversed for each of the movement elements (ie, higher scores representing better performance; 0 became 1, 1 became 0).

Pearson’s correlation coefficients were calculated to assess the relationship between patients’ skilled reaching, ARAT, and FM-UE scores at different time points poststroke. Significance levels were set at *P* = .05. All statistical analyses were performed using SPSS, Version 25.0.

## Results

### Subjects

Twelve patients were included. In 10 of these patients, the nondominant side was affected. Eleven patients were right-handed. Infarcts were subcortical, mixed cortical and subcortical, or in the brainstem, with lesion volumes ranging between 0.4 and 192 × 10^3^ mm^3^ (0.1% to 26.3% of the hemispheric volume). Table 1 shows the demographic and clinical characteristics.

**Table 1.** Patients’ Demographic and Clinical Characteristics at Enrolment.

Age, years; mean (SD)	58.3 (10.2)
Male/female	7/5
Handedness	11 right, 1 left
Time poststroke, days; mean (SD)	14.7 (3.6)
Lesion side	10 ND, 2 D
Stroke subtype	8 SC, 2 M, 2 B
Lesion volume, ×10^3^ mm^3^; mean (SD)^a^	21 (57)
FM-UE score; mean (SD)	18.6 (13.1)

Abbreviations: SD, standard deviation; MRI, magnetic resonance imaging; FM-UE, FM-UE: Fugl-Meyer Upper Extremity; D, dominant; ND, nondominant; SC, subcortical; M, mixed cortical and subcortical; B, brainstem.

a From 11 patients with MRI scan between 6 and 50 weeks poststroke.

From the preclinical study, 17 rats were included. Three of the 17 rats had to be euthanized prior to the end of the study (ie, before day 23) because of welfare issues due to severe weight loss or inner ear infection, resulting in missing data points. Rats all had stroke on their dominant (for reaching) side. Infarcts were located in the sensorimotor cortex (see Supplementary File C, Figure 1 for representative anatomical MR images; available online) and had a size of 22 ± 6 mm^3^ (2.9 ± 0.8% of the hemispheric volume). Table 2 shows the demographic and clinical characteristics.

**Table 2.** Rats’ Demographic and Clinical Characteristics.

Age, weeks^a^	10-11
Weight (g); mean (SD)^a^	326 (26)
Male/female	17/0
Lesion side	17 D
Stroke subtype	17 C
Lesion volume, mm^3^; mean (SD)^b^	22 (6)
SR success rate^c^, %; mean (SD)	17 (16)

Abbreviations: SD, standard deviation; MRI, magnetic resonance imaging; D, dominant; C, cortical; SR, skilled reaching.

a Age/weight at the time of stroke induction.

b From anatomical MRI scan at 17 days poststroke.

c At day 3 poststroke.

### Skilled Reaching Success Scores

**Figure 2.**
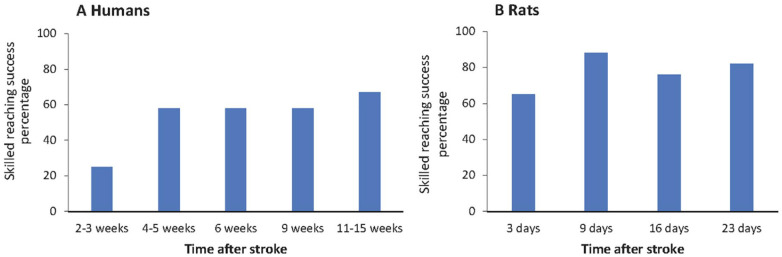
Success percentage of skilled reaching performance at different time points after stroke in humans (left) and rats (right). Note: Humans had a success percentage of 100% with the unaffected arm at each time point. Rats had a success percentage of 100% prestroke.

The percentages of patients that could successfully perform the skilled reaching task at the different time points poststroke are shown in Figure 2A. A quarter of the patients could successfully perform skilled reaching at 2 to 3 weeks poststroke (early subacute). The success percentage increased over time and was 67% at 11 to 15 weeks after stroke. The success percentage for skilled reaching with the unaffected arm was 100% at all time points.

The success percentages at different poststroke time points for the rats are shown in Figure 2B. The percentage of animals that executed at least one successful reach at the first early subacute time point (3 days poststroke) was 65%, which subsequently increased. At the final late subacute time point (23 days poststroke), 82% of the rats could successfully execute the skilled reaching task. The success percentage before stroke was 100%. The conventionally calculated success rate in rats, that is, the percentage of successfully obtained pellets with respect to the number of trials, was 38 ± 17% before stroke, dropped to 17 ± 16% at 3 days after stroke, and partially recovered to 25 ± 20% at day 23 poststroke (see Supplementary File C, Figure 2, available online).

### Movement (Sub)element Scores

**Figure 3.**
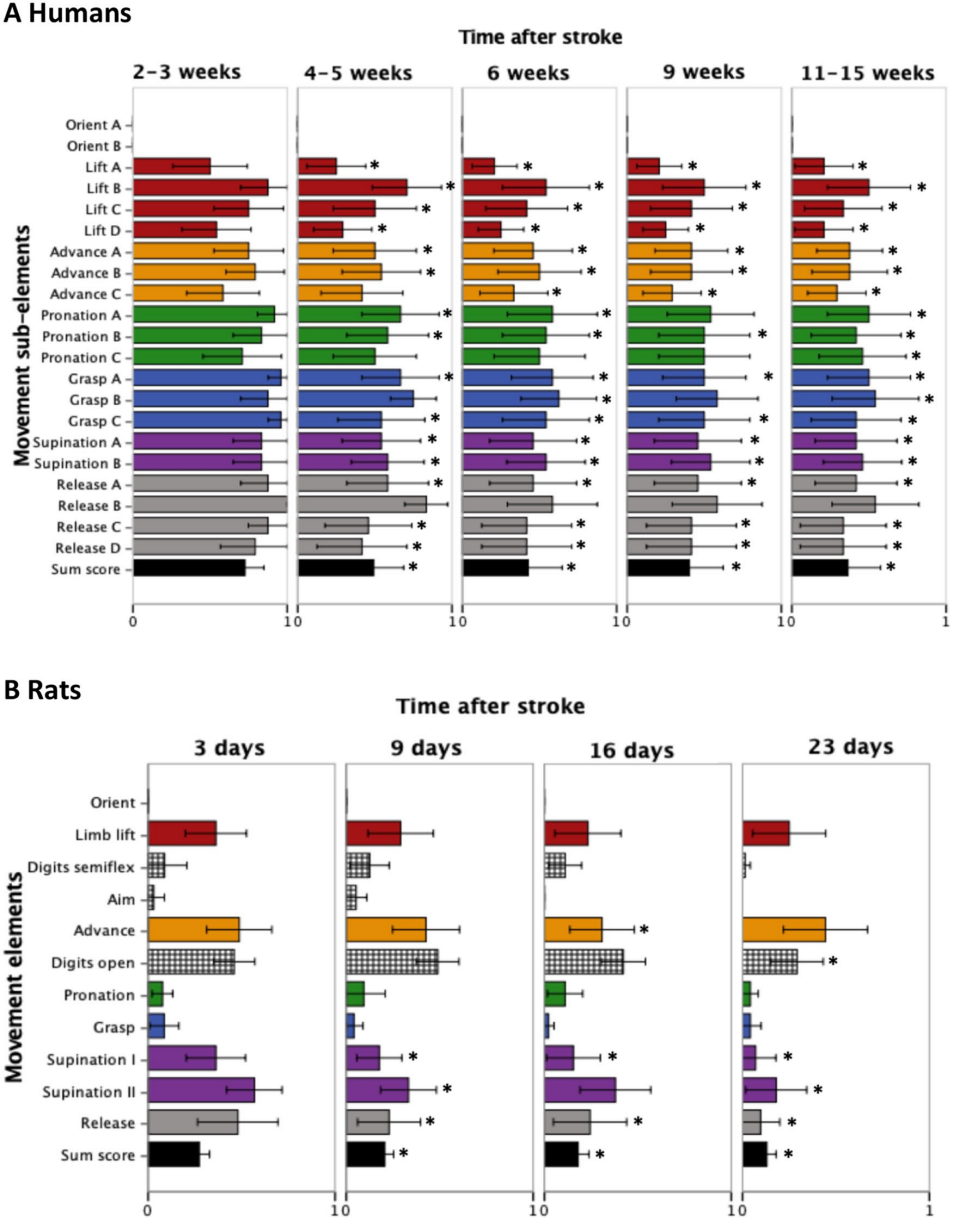
Movement (sub)element scores for patients (A) and rats (B) at different time points after stroke. Each bar represents the mean ± SD; non-colored elements, displayed as black-white blocked bars, are rat-specific elements; 0 = movement is present/normal; 0.5 = movement is present but incomplete; 1 = no movement; *Significant difference (*P* < .05).

Figure 3 shows the patient and rat scores for the individual movement (sub)elements during execution of the skilled reaching task at the different poststroke time points. At all poststroke time points, the movement element orient was unaffected in patients as well as rats, reflected by a score of 0 for the orient (sub)element(s). All other movement elements were affected (ie, incomplete or absent) as a result of the stroke, although scores were generally higher—reflective of a higher degree of deficiency—in patients than in rats.

At the second time point (4-5 weeks after stroke in patients; 9 days after stroke in rats) there were significant improvements for most movement elements in comparison to the first measurement (*P* < .05), except for the movement subelements advance C, pronation C, grasp B, and release B in patients (*P* > .05). In rats, there were significant improvements for the movement elements supination (I and II) and release at the second time point (*P* < .05), and additional improvements for the movement elements supination I and release were measured at subsequent time points.

From week 6 toward the last time point in patients, most movement elements, including subelements grasp B and advance C, showed significant improvement (*P* < .05). Movement subelements pronation C and release B, however, showed no significant improvement at weeks 6 and 9.

At the final time point (11-15 weeks after stroke in patients; 23 days after stroke in rats) all movement subelements in patients, except for release B, were significantly improved compared with the first time point (*P* < .05). In contrast, in rats only the movement elements digits open, supination I and II, and release were significantly improved at the final time point as compared with the first time point (*P* < .05).

Recovery of skilled reaching performance was also expressed by the change in the patients’ and rats’ skilled reaching overall sum score, which significantly improved between the first time point, and all subsequent time points (*P* < .05).

### Relationship Between Skilled Reaching and Clinical Outcome Measures in Stroke Patients

**Figure 4.**
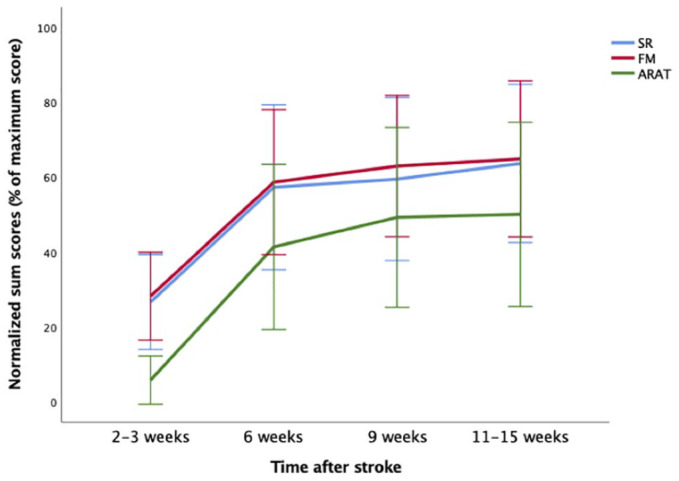
Relative sum scores (normalized to the maximum score of that particular test) from skilled reaching (SR), ARAT, and FM-UE assessments of stroke patients over time. Skilled reaching scores were reversed (ie, higher scores representing better performance) for comparison with the FM-UE and ARAT scores. Error bars represent standard error of the mean.

Figure 4 shows the time course of normalized skilled reaching, FM-UE, and ARAT sum scores for the stroke patients. For all 3 measures, the scores at the 6, 9, and 11 to 15 weeks were significantly improved in comparison to the scores at 2 to 3 weeks poststroke (skilled reaching: β = 11.53, SE = 2.18, *P* = .000; ARAT: β = 14.82, SE = 3.09, *P* = .000; and FM-UE: β = 11.88, SE = 2.11, *P* = .000.) (FM-UA assessment was not performed at 4-5 weeks poststroke, so we left out this time point for comparisons.) The normalized skilled reaching and FM-UE sum scores were highly similar at the individual time points (β = 1.75, SE = 6.12, *P* = .775), which was further emphasized by similar temporal recovery patterns (β = 0.10, SE = 3.27, *P* = .975). The normalized skilled reaching and ARAT sum scores differed significantly across separate time points poststroke (β = −19.11, SE = 6.13, *P* = .002). However, the temporal patterns of the normalized skilled reaching and ARAT sum scores were not significantly different (β = 2.46, SE = 3.31, *P* = .459).

Table 3 shows that skilled reaching, ARAT, and FM-UE sum scores were strongly correlated at all included time points poststroke (*P* < .05).

**Table 3.** Pearson’s Correlations Between Patients’ Skilled Reaching, ARAT, and FM-UE Sum Scores at Different Time Points After Stroke.

	Skilled reaching			
	2-3 weeks	6 weeks	9 weeks	11-15 weeks
ARAT	.647*	.888*	.871*	.963*
FM-UE	.717*	.917*	.911*	.966*

Abbreviations: ARAT, Action Research Arm Test; FM-UE, Fugl-Meyer Upper Extremity.

* *P* < .05.

## Discussion

Skilled reaching has been proposed as one of the most potent translational behavioral tests for studying poststroke recovery in rodents.^9^ Gradual improvement in skilled reaching performance after stroke has been previously reported for both humans^25^ and rodents.^26^ In the present study, we compared skilled reaching characteristics between rats and humans recovering from stroke, and we assessed the relation between skilled reaching performance and clinical outcome measures. Our study shows that skilled reaching performance follows a very comparable temporal pattern in humans and rodents during the subacute stages after stroke. Functional impairment was characterized by muscle flaccidity at the first measurement poststroke, prohibiting lift and advance movements. Subsequently, skilled reaching performance improved, but with a delay in motor recovery for distal muscles in the lower arm and hand (ie, pronation, grasp, and release). Patients’ skilled reaching scores showed strong correlations with clinical outcome measures (ARAT and FM-UE) at different time points during the first 3 months poststroke. The normalized skilled reaching overall sum scores matched with the normalized FM-UE sum scores, but were higher than the sum scores of an arm-specific measure of activity limitation (ARAT).

### Similarities and Differences in Poststroke Recovery of Skilled Reaching Performance Between Humans and Rats

It is known that the time course of stroke recovery in rodent models is more rapid than in human patients.^27-30^ Therefore, we chose to compare the first 3 months poststroke in human stroke patients to the first 3 to 4 weeks poststroke in rats, which is the typical period for reaching a plateau stage of motor recovery in these species, respectively.^15^

Success rate is the most commonly used outcome parameter to quantify functional deficits from a skilled reaching test. In rodent studies, success rates are typically calculated as the number of successful reaches divided by the number of trials, multiplied by 100%.^12^ In the current study, we also applied an adapted success percentage score, defined as the number of rats that could completely execute at least one successful reach, divided by the total number of rats, and multiplied by 100%, which was identical to the scoring system for the stroke patients. Success percentages for both humans and rats showed poststroke impairment in skilled reaching. The scores for human patients reflected more severe initial deficits, but a larger degree of subsequent recovery, as compared to the poststroke rats. This may be explained by differences in stroke severity and phase between the stroke patients and our rat stroke model, as further outlined below. In addition, humans had to grasp a smaller food item relative to hand size in comparison to rats, which may have made the task more challenging. While rats apply similar grasp patterns (whole paw grasp) for small and large food items, humans use pincer grasp for small items and precision grasp for larger items.^12^ Of note, variation in baseline skilled reaching abilities, which, for example, has been observed between different rat strains, may also contribute to differences in recovery patterns.^18^

Previous studies in rat models of stroke have shown that skilled reaching success rates may not expose remaining motor deficits,^26,31-33^ which may be better assessed by analysis of the skilled reaching movement elements. The movements that articulate skilled reaching displayed similar recovery patterns between humans and rats. This finding emphasizes the translational value of skilled reaching testing for preclinical and clinical research on stroke recovery. At the early subacute stage poststroke, muscle flaccidity could be observed in both species, which contributes to complications in the voluntary execution of movement elements, such as lift and advance. Body postural compensation, with stronger reliance on proximal muscles, was observed, and related functions, for example, lift, were less affected or showed rapid recovery. In human stroke patients, functional recovery of the distal muscles in the lower arm, responsible for pronation, grasp, and release, showed no (release B) or delayed improvement (pronation C and grasp B) in the late subacute phase poststroke. Correspondingly, in rats proximal movement elements (ie, limb lift, digits semi-flex, and aim) were less affected at the first subacute measurement, while distal movement elements (ie, supination, release) and particularly digit motor control (ie, digits open) still improved toward late subacute stages. These findings are in line with results from other studies that described recovery of proximal joint movements preceding recovery of distal movements, suggesting differences in neural substrates for paretic upper limb recovery.^22,34,35^ In our study, the relative infarct size in rats was within the range of relative infarct sizes in stroke patients. Hemispheric infarct volume was about 3% in rats and ranged between 0.1% and 26% in humans. However, in contrast to the variety in lesion location in patients, the stroke lesion in rats was confined to the sensorimotor cortex, leaving a large part of the sensorimotor system intact. This may have facilitated the progression of recovery. Nevertheless, several movement elements (eg, partial rotation) remained impaired at the final measurement poststroke, consistent with an earlier study in the same rodent stroke model.^26^ Delayed or incomplete recovery of these movement elements could be a consequence of infarction to a relatively large part of the motor cortex.^36,37^ This contrasts with other commonly used behavioral measures of sensorimotor function (eg, forelimb or hindlimb placing ability) that often show complete recovery within 2 to 4 weeks after stroke, which emphasizes the sensitivity of the skilled reaching test.^38^

The human stroke patients in our study, who had varying infarct locations, also showed significant persistence of upper limb impairment in the first 3 months poststroke, which is in accordance with previous studies.^39,40^ The persistence of impairment in human and rat skilled reaching movements suggests that some recovery could have been achieved through use of compensatory strategies.

### Relation Between Skilled Reaching Performance and Clinical Outcome Measures

Our study showed strong positive correlations between the normalized sum scores of skilled reaching and clinical outcome measures from the ARAT and FM-UE assessment at each of the time points during the first 3 months poststroke. The 3 tests share measurement of reach and lift components, and a lower level of impairment on these elements will be reflected in better (functional) performance across a broad range of movements.^41,42^ In addition, the design of the motor section of the FM-UE assessment is based on synergies, that is, systematic coupling across different joints or a fixed pattern of co-activation of muscles, of which disruptions result in reaching deficits.^41,43^ Importantly, because there are no equivalents of the ARAT or FM-UE assessment for rodents, the correlation between skilled reaching performance and these clinical outcome measures highlight the significance of skilled reaching assessment in the alignment of preclinical and clinical stroke research.

Consistent with previous research, skilled reaching scores and clinical outcome scores improved significantly over the 3-month period poststroke.^11^ The largest improvements were seen in the first 5 to 6 weeks poststroke. In general, spontaneous neurological recovery progresses fastest in the first months poststroke, after which recovery levels off and reaches a plateau after 3 to 6 months.^44,45^ Interestingly, the ARAT total scores were significantly different from the skilled reaching total scores. The ARAT, which assesses activity limitation, presented lower normalized baseline scores compared to the skilled reaching and FM-UE tests. Features of the measures, such as the representation of specific parts of the upper limb within the individual measures, may underlie the differences in scores. In the ARAT, 16% of the total score is obtained from assessment of gross arm movements (ie, placement of hand behind head, on top of head, and to the mouth), while the majority of the score is based on movements that require some form of (finer) hand and digit motor control. Fine motor control involves motor coordination, speed of movement, and force scaling.^46^ In contrast, in skilled reaching testing the first relatively gross movement elements (orient, lift, advance, and pronation) make up 57% of the total score, whereas grasp, which requires fine hand and digit motor control, constitutes 43% of the total score.^18,47^ Our findings demonstrate that motor recovery follows a proximal-distal principle, which is in agreement with Twitchell and Brunnstrom’s concept of sequential return of motor function in the hemiplegic stroke patient, where hand and digit motor control recover at later stages.^22^ The underrepresentation of distal fine motor function may explain the higher scores on the FM-UE and skilled reaching tests as compared with the ARAT scores in the subacute period poststroke.

One of the strengths of the skilled reaching task is its ability to allow for distinction between compensation and recovery.^15^ This is in contrast with the ARAT, which is a performance-based measure focused on task accomplishment, with little regard for how the task is accomplished. The ARAT therefore precludes distinguishing between true motor recovery and compensatory movement strategies.^6^ The FM-UE assessment is largely immune to compensation and can be valuable for monitoring true recovery of motor functions. However, the FM-UE test is infrequently used today in clinical practice, as it is a time-consuming measure to administer.^41^

In the past 40 years, clinical priority shifted away from impairment-oriented training toward training of activities of daily living with functional tasks, since relearning normal patterns of movement did not inevitably generalize to activities of daily living.^41,48^ Our current findings suggest that restoration of motor function is still feasible up to at least 3 months poststroke. This implies that impairment-oriented training could still be relevant in motor rehabilitation, and underscores the significance of incorporating measures at the impairment level to distinguish between true motor recovery and compensation after stroke in (pre)clinical research and in clinical practice.

### Limitations

Although our study enabled direct translation of poststroke skilled reaching performance assessments between rats and humans after stroke, there were some limitations. First, we could not consider the impact of handedness on outcome measures, since only 2 patients had an affected dominant upper limb. While rats were trained to use their dominant/preferred forelimb to reach for the target, humans were instructed to use their affected limb, which was not always the dominant one. Second, we did not assess other factors, such as vision, olfaction, fatigue, or self-efficacy, which may have contributed to reach-to-eat performance in rats and patients. Third, no kinematic assessment was performed, which could have improved the sensitivity to capture subtle movement qualities and compensatory motions.^49^ Fourth, patients and rats in our study were subjects in ongoing clinical and preclinical trials, respectively, in which the effect of rTMS on motor recovery is investigated. The intervention may have impacted the recovery profiles.

## Conclusions

This study shows that skilled reaching performance improves significantly over time in humans and rats recovering from stroke. Both species showed muscle flaccidity in the early subacute phase, early recovery of proximal movements, and a delayed motor recovery of distal muscles in the lower arm/forelimb. The recovery of skilled reaching performance in human stroke patients strongly resembled the recovery patterns of commonly used clinical outcome measures from the ARAT and FM-UE assessment, which underlines the translational significance of the skilled reaching task in preclinical research. Furthermore, skilled reaching assessment can serve as a complementary tool to distinguish between recovery and compensation in clinical care.

## Supplemental Material

sj-docx-1-nnr-10.1177_15459683211005022 – Supplemental material for Translational Value of Skilled Reaching Assessment in Clinical and Preclinical Studies on Motor Recovery After Stroke Click here for additional data file. Supplemental material, sj-docx-1-nnr-10.1177_15459683211005022 for Translational Value of Skilled Reaching Assessment in Clinical and Preclinical Studies on Motor Recovery After Stroke by Eline C. C. van Lieshout, Julia Boonzaier, Adam J. Pel, Caroline L. van Heijningen, Jord J. Vink, Johanna M. A. Visser-Meily, Geralda A. F. van Tilborg and Rick M. Dijkhuizen in Neurorehabilitation and Neural Repair

sj-docx-2-nnr-10.1177_15459683211005022 – Supplemental material for Translational Value of Skilled Reaching Assessment in Clinical and Preclinical Studies on Motor Recovery After Stroke Click here for additional data file. Supplemental material, sj-docx-2-nnr-10.1177_15459683211005022 for Translational Value of Skilled Reaching Assessment in Clinical and Preclinical Studies on Motor Recovery After Stroke by Eline C. C. van Lieshout, Julia Boonzaier, Adam J. Pel, Caroline L. van Heijningen, Jord J. Vink, Johanna M. A. Visser-Meily, Geralda A. F. van Tilborg and Rick M. Dijkhuizen in Neurorehabilitation and Neural Repair

sj-docx-3-nnr-10.1177_15459683211005022 – Supplemental material for Translational Value of Skilled Reaching Assessment in Clinical and Preclinical Studies on Motor Recovery After Stroke Click here for additional data file. Supplemental material, sj-docx-3-nnr-10.1177_15459683211005022 for Translational Value of Skilled Reaching Assessment in Clinical and Preclinical Studies on Motor Recovery After Stroke by Eline C. C. van Lieshout, Julia Boonzaier, Adam J. Pel, Caroline L. van Heijningen, Jord J. Vink, Johanna M. A. Visser-Meily, Geralda A. F. van Tilborg and Rick M. Dijkhuizen in Neurorehabilitation and Neural Repair

## References

[1] Lawrence ES Coshall C Dundas R, et al. Estimates of the prevalence of acute stroke impairments and disability in a multiethnic population. Stroke. 2001;32:1279-1284. doi:10.1161/01.STR.32.6.1279 11387487

[2] Lee KB Lim SH Kim KH, et al. Six-month functional recovery of stroke patients: a multi-time-point study. Int J Rehabil Res. 2015;38:173-180. doi:10.1097/MRR.0000000000000108 25603539 PMC4415968

[3] Nichols-Larsen DS Clark PC Zeringue A Greenspan A Blanton S. Factors influencing stroke survivors’ quality of life during subacute recovery. Stroke. 2005;36:1480-1484. doi:10.1161/01.STR.0000170706.13595.4f 15947263

[4] Foroud A Whishaw IQ. Changes in the kinematic structure and non-kinematic features of movements during skilled reaching after stroke: a Laban movement analysis in two case studies. J Neurosci Methods. 2006;158:137-149. doi:10.1016/j.jneumeth.2006.05.007 16766042

[5] Taub E Uswatte G Mark VW. The functional significance of cortical reorganization and the parallel development of CI therapy. Front Hum Neurosci. 2014;8:396. doi:10.3389/fnhum.2014.00396 25018720 PMC4072972

[6] Levin MF Kleim JA Wolf SL. What do motor “recovery” and “compensation” mean in patients following stroke? Neurorehabil Neural Repair. 2008;23:313-319. doi:10.1177/1545968308328727 19118128

[7] Jones T. Motor compensation and its effects on neural reorganization after stroke. Nat Rev Neurosci. 2017;18:276-278. doi:10.1002/14651858.CD002840 PMC6289262 28331232

[8] Corbett D Carmichael ST Murphy TH, et al. Enhancing the alignment of the preclinical and clinical stroke recovery research pipeline: consensus-based core recommendations from the Stroke Recovery and Rehabilitation Roundtable translational working group. Int J Stroke. 2017;12:462-471. doi:10.1177/1747493017711814 28697710

[9] Klein A Sacrey LAR Whishaw IQ Dunnett SB. The use of rodent skilled reaching as a translational model for investigating brain damage and disease. Neurosci Biobehav Rev. 2012;36:1030-1042. doi:10.1016/j.neubiorev.2011.12.010 22227413

[10] Whishaw IQ Pellis SM. The structure of skilled forelimb reaching in the rat: a proximally driven movement with a single distal rotatory component. Behav Brain Res. 1990;41:49-59. doi:10.1016/0166-4328(90)90053-H 2073355

[11] Sacrey LAR Alaverdashvili M Whishaw IQ. Similar hand shaping in reaching-for-food (skilled reaching) in rats and humans provides evidence of homology in release, collection, and manipulation movements. Behav Brain Res. 2009;204:153-161. doi:10.1016/j.bbr.2009.05.035 19520119

[12] Whishaw IQ Pellis SM Gorny BP. Skilled reaching in rats and humans: evidence for parallel development or homology. Behav Brain Res. 1992;47:59-70. doi:10.1016/S0166-4328(05)80252-9 1571101

[13] Alaverdashvili M Whishaw IQ. A behavioral method for identifying recovery and compensation: hand use in a preclinical stroke model using the single pellet reaching task. Neurosci Biobehav Rev. 2013;37:950-967. doi:10.1016/j.neubiorev.2013.03.026 23583614

[14] Balkaya M Cho S. Optimizing functional outcome endpoints for stroke recovery studies. J Cereb Blood Flow Metab. 2019;39:2323-2342. doi:10.1177/0271678X19875212 31522590 PMC6893977

[15] Krakauer JW Carmichael ST Corbett D Wittenberg GF. Getting neurorehabilitation right: what can be learned from animal models? Neurorehabil Neural Repair. 2012;26:923-931. doi:10.1177/1545968312440745 22466792 PMC4554531

[16] van Lieshout ECC Visser-Meily JMA Neggers SFW van der Worp HB Dijkhuizen RM. Brain stimulation for arm recovery after stroke (B-STARS): protocol for a randomised controlled trial in subacute stroke patients. BMJ Open. 2017;7:e016566. doi:10.1136/bmjopen-2017-016566 PMC5629737 28851789

[17] McDonald MW Hayward KS Rosbergen ICM Jeffers MS Corbett D. Is environmental enrichment ready for clinical application in human post-stroke rehabilitation? Front Behav Neurosci. 2018;12:135. doi:10.3389/fnbeh.2018.00135 30050416 PMC6050361

[18] Whishaw IQ Gorny B Foroud A Kleim JA. Long-Evans and Sprague-Dawley rats have similar skilled reaching success and limb representations in motor cortex but different movements: some cautionary insights into the selection of rat strains for neurobiological motor research. Behav Brain Res. 2003;145:221-232. doi:10.1016/S0166-4328(03)00143-8 14529819

[19] Eshkol N Wachmann A. Movement Notation. Weidenfeld & Nicolson; 1958.

[20] Duncan PW Propst M Nelson SG. Reliability of the Fugl-Meyer assessment of sensorimotor recovery following cerebrovascular accident. Phys Ther. 1983;63:1606-1610. doi:10.1590/S1806-64452009000200004 6622535

[21] Fugl-Meyer AR Jääskö L Leyman I Olsson S Steglind S. The post stroke hemiplegic patient. I. A method for evaluation of physical performance. Scand J Rehabil Med. 1975;7:13-31. 1135616

[22] Gladstone DJ Danells CJ Black SE. The Fugl-Meyer assessment of motor recovery after stroke: a critical review of its measurement properties. Neurorehabil Neural Repair. 2002;16:232-240. doi:10.1177/154596802401105171 12234086

[23] van der Lee JH Roorda LD Beckerman H Lankhorst GJ Bouter LM. Improving the Action Research Arm test: a unidimensional hierarchical scale. Clin Rehabil. 2002;16:646-653. doi:10.1191/0269215502cr534oa 12392340

[24] Field A Hole G. How to Design and Report Experiments. Sage; 2003.

[25] Foroud A Whishaw IQ. Reaching-to-eat in humans post-stroke: fluctuating components within a constant pattern. Behav Neurosci. 2010;124:851-867. doi:10.1037/a0021112 21133536

[26] Moon SK Alaverdashvili M Cross AR Whishaw IQ. Both compensation and recovery of skilled reaching following small photothrombotic stroke to motor cortex in the rat. Exp Neurol. 2009;218:145-153. doi:10.1016/j.expneurol.2009.04.021 19409894

[27] Langhorne P Bernhardt J Kwakkel G. Stroke rehabilitation. Lancet. 2011;377:1693-1702. doi:10.1016/S0140-6736(11)60325-5 21571152

[28] Kwakkel G Winters C van Wegen EEH, et al. Effects of unilateral upper limb training in two distinct prognostic groups early after stroke: the EXPLICIT-stroke randomized clinical trial. Neurorehabil Neural Repair. 2016;30:804-816. 26747128 10.1177/1545968315624784

[29] Winters C Van Wegen EEH Daffertshofer A Kwakkel G. Generalizability of the proportional recovery model for the upper extremity after an ischemic stroke. Neurorehabil Neural Repair. 2015;29:614-622. doi:10.1177/1545968314562115 25505223

[30] Sengupta P. The laboratory rat: relating its age with human’s. Int J Prev Med. 2013;4:624-630. 23930179 PMC3733029

[31] Alaverdashvili M Foroud A Lim DH Whishaw IQ. “Learned baduse” limits recovery of skilled reaching for food after forelimb motor cortex stroke in rats: a new analysis of the effect of gestures on success. Behav Brain Res. 2008;188:281-290. doi:10.1016/j.bbr.2007.11.007 18155782

[32] Metz GA Antonow-Schlorke I Witte OW. Motor improvements after focal cortical ischemia in adult rats are mediated by compensatory mechanisms. Behav Brain Res. 2005;162:71-82. doi:10.1016/j.bbr.2005.03.002 15922067

[33] Gharbawie OA Whishaw IQ. Parallel stages of learning and recovery of skilled reaching after motor cortex stroke: “oppositions” organize normal and compensatory movements. Behav Brain Res. 2006;175:249-262. doi:10.1016/j.bbr.2006.08.039 17049628

[34] Luke C Dodd KJ Brock K. Outcomes of the Bobath concept on upper limb recovery following stroke. Clin Rehabil. 2004;18:888-898. doi:10.1191/0269215504cr793oa 15609844

[35] Schambra HM Xu J Branscheidt M, et al. Differential poststroke motor recovery in an arm versus hand muscle in the absence of motor evoked potentials. Neurorehabil Neural Repair. 2019;33:568-580. doi:10.1177/1545968319850138 31170880 PMC6631316

[36] Whishaw IQ Pellis SM Gorny BP Pellis VC. The impairments in reaching and the movements of compensation in rats with motor cortex lesions: an endpoint, video recording, and movement notation analysis. Behav Brain Res. 1991;42:77-91. doi:10.1016/S0166-4328(05)80042-7 2029348

[37] Whishaw IQ. Loss of the innate cortical engram for action patterns used in skilled reaching and the development of behavioral compensation following motor cortex lesions in the rat. Neuropharmacology. 2000;39:788-805. doi:10.1016/S0028-3908(99)00259-2 10699445

[38] Madinier A Quattromani MJ Sjölund C Ruscher K Wieloch T. Enriched housing enhances recovery of limb placement ability and reduces aggrecan-containing perineuronal nets in the rat somatosensory cortex after experimental stroke. PLoS One. 2014;9:e93121. doi:10.1371/journal.pone.0093121 PMC3963994 24664200

[39] Kwakkel G Kollen BJ van der Grond JV Prevo AJH. Probability of regaining dexterity in the flaccid upper limb: impact of severity of paresis and time since onset in acute stroke. Stroke. 2003;34:2181-2186. doi:10.1161/01.STR.0000087172.16305.CD 12907818

[40] Brunner IC Skouen JS Strand LI. Recovery of upper extremity motor function post stroke with regard to eligibility for constraint-induced movement therapy. Top Stroke Rehabil. 2011;18:248-257. doi:10.1310/tsr1803-248 21642062

[41] Krakauer JW Carmichael ST. Broken Movement: The Neurobiology of Motor Recovery After Stroke. MIT Press; 2017. doi:10.7551/mitpress/9310.001.0001

[42] Nijland RHM van Wegen EEH Harmeling-van der Wel BC Kwakkel G; EPOS Investigators. Presence of finger extension and shoulder abduction within 72 hours after stroke predicts functional recovery: early prediction of functional outcome after stroke: the EPOS cohort study. Stroke. 2010;41:745-750. doi:10.1161/STROKEAHA.109.572065 20167916

[43] Balbinot G Schuch CP Jeffers MS McDonald MW Livingston-Thomas JM Corbett D. Post-stroke kinematic analysis in rats reveals similar reaching abnormalities as humans. Sci Rep. 2018;8:8738. doi:10.1038/s41598-018-27101-0 29880827 PMC5992226

[44] Krakauer JW Marshall RS. The proportional recovery rule for stroke revisited. Ann Neurol. 2015;78:845-847. doi:10.1002/ana.24537 26435166

[45] Schaechter JD. Motor rehabilitation and brain plasticity after hemiparetic stroke. Prog Neurobiol. 2004;73:61-72. doi:10.1016/j.pneurobio.2004.04.001 15193779

[46] Allgöwer K Hermsdörfer J. Fine motor skills predict performance in the Jebsen Taylor Hand Function Test after stroke. Clin Neurophysiol. 2017;128:1858-1871. doi:10.1016/j.clinph.2017.07.408 28826016

[47] Lang CE Wagner JM Dromerick AW Edwards DF. Measurement of upper-extremity function early after stroke: properties of the action research arm test. Arch Phys Med Rehabil. 2006;87:1605-1610. doi:10.1016/j.apmr.2006.09.003 17141640

[48] Carr JH. Movement Science: Foundations for Physical Therapy in Rehabilitation. Aspen; 1987.

[49] Bernhardt J Hayward KS Kwakkel G, et al. Agreed definitions and a shared vision for new standards in stroke recovery research: the Stroke Recovery and Rehabilitation Roundtable taskforce. Int J Stroke. 2017;12:444-450. doi:10.1177/1747493017711816 28697708

