# Supplementary material for: Translational Value of Skilled Reaching Assessment in Clinical and Preclinical Studies on Motor Recovery After Stroke
Source: Neurorehabil Neural Repair. 2021 Apr 7;35(5):457–67. doi: 10.1177/15459683211005022 (PMC8127668; doi:10.1177/15459683211005022)
Supplement: sj-docx-1-nnr-10.1177_15459683211005022 – Supplemental material for Translational Value of Skilled Reaching Assessment in Clinical and Preclinical Studies on Motor Recovery After Stroke [file sj-docx-1-nnr-10.1177_15459683211005022.docx]

**Supplementary File A**

# Housing (rats)

Rats were housed per 2 or 3 in standard cages (30 x 40 x 20 cm^3^), during the skilled pellet reaching training phase, up until day 2 after stroke (see **Figure 1**). The standard cages were equipped with bedding material, cage enrichment (orange rectangular Perplex tube) and nesting material, such as tissue paper.

Three days after stroke induction, the animals were moved to an enriched environment cage that was larger (70 x 60 x 50 cm^3^), contained multiple replaceable toys, platforms and ladders (changed twice per week), and housed 4-5 rats. This enriched environment represented a clinical rehabilitation setting.

# Skilled pellet reaching (rats)

## Food restriction:

Once the animals (6-weeks old) arrived at the animal facility, they were allowed to acclimatize to their new environment for five days under normal housing conditions and with ad libitum access to food and water (see **Figure 1**). Following acclimatization, animals were weighed and placed on food restriction to maintain 90 – 95% of their initial body weight. The animals, housed in pairs, received 26 – 30 grams of chow per day and water ad libitum. The food restriction functioned as a motivator for the animals to learn and to participate in the skilled pellet reaching task.

During the first week of food restriction, the animals also received a few sugar pellets (45 mg sucrose, unflavored; Dustless Precision Pellets, Bio-Serve®) with their chow to get accustomed to the taste. Following this first week, the animals started with the pre-training phase. The body weight of the animals was closely monitored throughout the entire experiment.


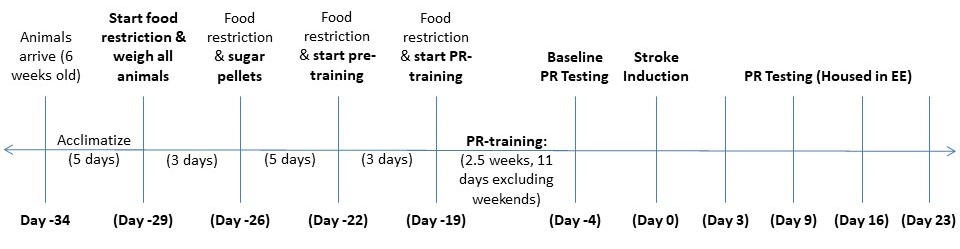


***Figure 1: Timeline overview of skilled pellet reaching training and testing.*** *After baseline skilled pellet reaching (PR) testing, animals received food and water ad libitum. Following PR testing on Day 3 the animals were moved into an enriched environment (EE).*

## Pre-training:

During pre-training, the goal was to stimulate the grasping of sugar pellets through the slot and to determine the dominant/preferred forelimb of each animal. The rats were placed in a Plexiglas box with multiple vertical slots in the front panel of the box (**Figure 2**). A plastic cup, filled with sugar pellets to the brim, was placed in front of the vertical slots. The animals were allowed to grasp as many sugar pellets as they could within a 30-minute period. During this period, the grasping behavior of the animals was observed in order to determine whether they had a preferred grasping-paw. After three consecutive days of pre-training a preferred forelimb could be identified and this was regarded as the dominant paw.


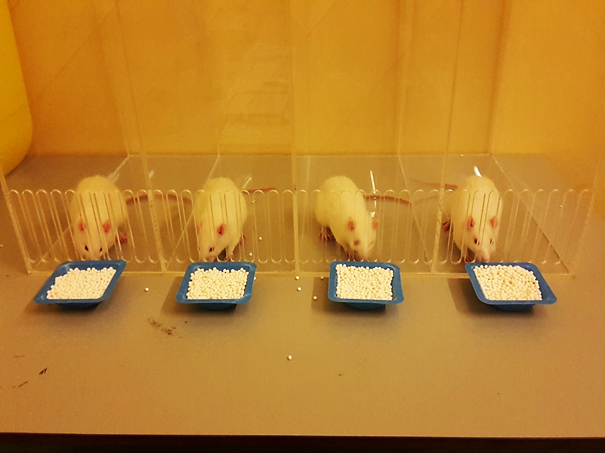


**Figure 2: Skilled pellet reaching pre-training box.** Plexiglas training boxes allowed for simultaneous training of multiple animals.

## Training:

Following the pre-training step, each individual rat was trained to perform the skilled pellet reaching task in one of two custom-built, clear, Plexiglas boxes (**Figure 3**). Both training boxes allowed the same training (no difference in training effect was observed between the two boxes (data not shown)), however one box was specifically designed for unobstructed high-speed recording of the grasping movement from the lateral aspect of the box (**Figure 3A**). In each of the training boxes, there were one or two vertical slots in the front panel of the box. At the bottom of each vertical slot, there was a shelf with an indentation that allowed pellet placement slightly off-center to the opening of the slot. The off-center positioning of the pellet was necessary to force the rats to use their ‘dominant’ forelimb to grasp the sugar pellet. The slot ipsilateral to the side of the preferred paw was used to train the animal (**Figure 3A; n = 5**). In the other skilled pellet reaching box (**Figure 3B; n = 12**), the sugar pellet was positioned on the indent contralateral to the preferred paw to train the animal.

Skilled pellet reaching training was performed for a maximum period of 15 minutes per day, Monday through Friday, for 2.5 weeks in one of the respective training boxes. The animals were trained to approach the shelf at the front of the box, reach for a pellet, and then turn around to walk to the rear of the box. If the rat succeeded to grasp the pellet from the shelf, it was considered a successful trial. Before a new trial could start, the animal first had to walk to the rear end of the box again before a new pellet could be obtained. If the rat knocked the pellet off of its original position, the pellet was removed from the shelf and it was regarded as a failed attempt.


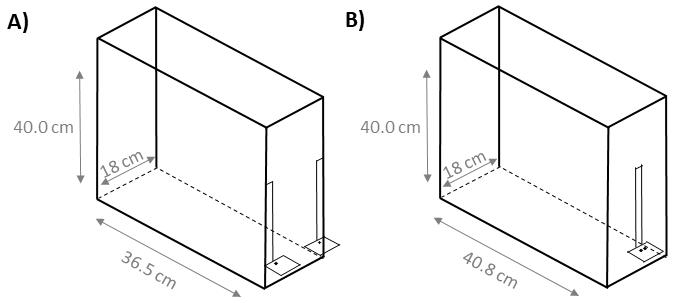


**Figure 3: Skilled pellet reaching boxes.** (A) Training box with two vertical slots of 1 cm wide, which extended 3 cm above the floor, located at the left and right end of the front side. At the bottom of the slots, there was a 3 cm wide shelf with an indentation that allowed pellet placement slightly lateral to the opening of the slot. To make sure the rat approached the correct slot, the other slot was blocked with a metal object.

*(B) Training box with a single vertical slot of 1 cm wide in the center of the front side, which extended 3 cm above the floor. At the bottom of the vertical slot there was a 4.5 cm wide shelf with two indentations that allowed for the placement and grasping of a sugar pellet from the indent contralateral to the preferred paw.*

## Data acquisition:

On the behavioral testing days the skilled pellet reaching task was recorded from the frontal plane using a Panasonic HC V520 camera (50 fps). These recordings were used for quantitative and qualitative pellet reaching analyses. During the testing sessions light was provided from two light sources in the direction that was filmed.

# Photothrombotic stroke induction (rats)

A photothrombotic stroke was induced in the hemisphere contralateral to the preferred forelimb (Watson *et al.*, 1985; Schmidt *et al.*, 2012). Pre-operatively, all rats received 5 mg/kg carprofen subcutaneously to minimize pain during and after surgery. Rats were anesthetized with isoflurane (5% induction, 2% maintenance in air/O_2_ (4/1)), intubated for mechanical ventilation, and placed in a stereotaxic frame. A feedback-controlled heating pad ensured that the body temperature of the animals was maintained at 37.0 ± 1.0 °C. After shaving of the head, the skull was disinfected with 70% alcohol and a midline incision was made along the scalp. Xylocaine (Lidocaine spray 100 mg/ml, AstraZeneca B.V. Louis, Zoetermeer) was applied in the incision for local analgesia. Using a scalpel, the periosteum was scraped away and the skull was dried with sterile cotton swabs. The coordinates of bregma were determined using a stereotact and the sensorimotor cortex was identified as a rectangular section 1.5 to 4.5 mm lateral to bregma, and 4.0 to -4.0 mm anterior-posterior to bregma. Black tape was used to mask the skull outside of the region of interest. Next, Rose Bengal (25 mg/ml, Sigma Aldrich/Merck, The Netherlands), a photosensitive dye, was injected intravenously via the vena saphena, and the brain was illuminated through the skull for 20 minutes, using a cold light source with a green fluorescent filter (Schott KL 1500 LCD, Germany). Following illumination, the scalp was sutured and the animal was placed in a heated cage to recover.

# Repetitive transcranial magnetic stimulation (rats)

From days six to sixteen post-stroke, the rats received repetitive transcranial magnetic stimulation (rTMS) treatment. This excluded weekend days, resulting in a total of nine treatment days. The stimulation protocols were executed with a 25-mm figure-of-eight TMS coil (Boonzaier *et al.*, 2019), while animals were anesthetized with isoflurane (1.5%). Prior to the start of rTMS treatment, the resting motor threshold was determined for each rat. This was done by stimulating the forelimb region of the brain with single TMS pulses, while simultaneously recording muscle activity of the musculus brachialis with electromyography (based on the protocol described in Boonzaier *et al.*, 2019). During stimulation, both the TMS coil and the animals were fixed in a stereotaxic frame to allow for accurate coil placement and stimulation. The animals either received high-frequency (5 Hz), low-frequency (1 Hz) or sham stimulation. Repetitive TMS treatment was given at 85% of the rat’s resting motor threshold for the high- and low-frequency treatment groups, whereas stimulation for the sham group was applied at 10% of the resting motor threshold intensity. Each rTMS treatment session lasted for 20 minutes.

*MRI*

Patients:

MRI scans were acquired at the University Medical Center Utrecht using a Philips 3T Achieva scanner. 3D T_1_-weighted anatomical MRI scans for lesion delineation were acquired at 5-6 weeks (n = 7), 3 months (n = 1), 6 months (n = 1) and/or 12 months (n = 2) after stroke. Anatomical MRI parameters were: repetition time (TR) = 8.13 ms, echo time (TE) = 3.7 ms, flip angle = 8°, field-of-view (FOV) = 512 × 512, spatial resolution: 0.47 × 0.47 × 1.0 mm^3^ (total scan time = 2.5 min). The images were resized to an isotropic voxel size of 1 mm^3^ before manual delineation of the lesions. SPM12 was used to inverse normalize a cerebral mask in MNI space to individual patient space in order to calculate individual cerebral hemispheric volumes.

Rats:

MRI was executed on a 9.4 T preclinical MR system (Varian Inc., Palo Alto, CA, USA) while animals were mechanically ventilated with 1.5% isoflurane in air/O_2_ (4:1). Anatomical images were acquired with a balanced steady-state free precession sequence (250 µm isotropic spatial resolution, TR = 5 ms, TE = 2.5 ms, flip angle = 20°, FOV = 40 × 32 × 24 mm^3^, matrix = 160 × 128 × 96, 3 averages and pulse angle shifts of 0°, 90°, 180° and 270°, total scan time = 12 min). The stroke infarction volume was calculated from manual segmentations of lesioned tissue.

**References**

Boonzaier J, Petrov PI, Otte WM, Smirnov N, Neggers SFW, Dijkhuizen RM. Design and Evaluation of a Rodent-Specific Transcranial Magnetic Stimulation Coil: An In Silico and In Vivo Validation Study. Neuromodulation. 2020 Apr;23(3):324-334. doi: 10.1111/ner.13025

.

Schmidt A, Hoppen M, Strecker JK, Diederich K, Schäbitz WR, Schilling M, Minnerup J. Photochemically induced ischemic stroke in rats. Exp Transl Stroke Med. 2012 Aug 9;4(1):13. doi: 10.1186/2040-7378-4-13.

Watson BD, Dietrich WD, Busto R, Wachtel MS, Ginsberg MD. Induction of reproducible brain infarction by photochemically initiated thrombosis. Ann Neurol. 1985 May;17(5):497-504. doi: 10.1002/ana.410170513.
