# Supplementary material for: Translational Value of Skilled Reaching Assessment in Clinical and Preclinical Studies on Motor Recovery After Stroke
Source: Neurorehabil Neural Repair. 2021 Apr 7;35(5):457–67. doi: 10.1177/15459683211005022 (PMC8127668; doi:10.1177/15459683211005022)
Supplement: sj-docx-2-nnr-10.1177_15459683211005022 – Supplemental material for Translational Value of Skilled Reaching Assessment in Clinical and Preclinical Studies on Motor Recovery After Stroke [file sj-docx-2-nnr-10.1177_15459683211005022.docx]

**Supplementary File B**

Table 1. Movement element rating scale for humans. From Klein et al. (2012).

| Element | Description |
| --- | --- |
| Limb lift | ﻿The head and the snout are oriented towards the target so that that rat sniffs the target |
| Digits semi flexed | ﻿The forepaw is lifted from the floor until the digits are aligned with the midline of the body |
| Aim | ﻿ The elbow is adducted to the body midline with a movement of the upper arm while the digits remain positioned on the body midline. |
| Advance | ﻿The forelimb moves forward through the slot |
| Digits open | ﻿The digits are extended as the limb is advanced and then are opened as the paw is pronated over the food |
| Pronation | ﻿The elbow abducts with a movement of the upper arm pronating the paw over the target in an arpeggio movement |
| Grasp | ﻿The arm remains still, while the digits close to grasp the food and then the paw is extended and raised |
| Supination I | ﻿The paw is supinated so that the palm faces the mouth |
| Supination II | ﻿The paw is supinated so that the palm faces the mouth |
| Release | ﻿The food pellet is released into the mouth by opening the digits |

Table 2. Movement element rating scale for rodents. From Moon et al. (2009).

**References**

Klein A, Sacrey LAR, Whishaw IQ, Dunnett SB. The use of rodent skilled reaching as a translational model for investigating brain damage and disease. Neurosci Biobehav Rev [Internet]. 2012;36(3):1030–42. Available from: http://dx.doi.org/10.1016/j.neubiorev.2011.12.010

Moon SK, Alaverdashvili M, Cross AR, Whishaw IQ. Both compensation and recovery of skilled reaching following small photothrombotic stroke to motor cortex in the rat. Exp Neurol [Internet]. 2009;218(1):145–53. Available from: http://dx.doi.org/10.1016/j.expneurol.2009.04.021
