# Supplementary material for: Translational Value of Skilled Reaching Assessment in Clinical and Preclinical Studies on Motor Recovery After Stroke
Source: Neurorehabil Neural Repair. 2021 Apr 7;35(5):457–67. doi: 10.1177/15459683211005022 (PMC8127668; doi:10.1177/15459683211005022)
Supplement: sj-docx-3-nnr-10.1177_15459683211005022 – Supplemental material for Translational Value of Skilled Reaching Assessment in Clinical and Preclinical Studies on Motor Recovery After Stroke [file sj-docx-3-nnr-10.1177_15459683211005022.docx]

**Supplementary File C**

*Photothrombotic stroke lesion*

Multislice MR images of post-stroke rat brain displayed a focal unilateral lesion in the sensorimotor cortex (**Figure 1**).


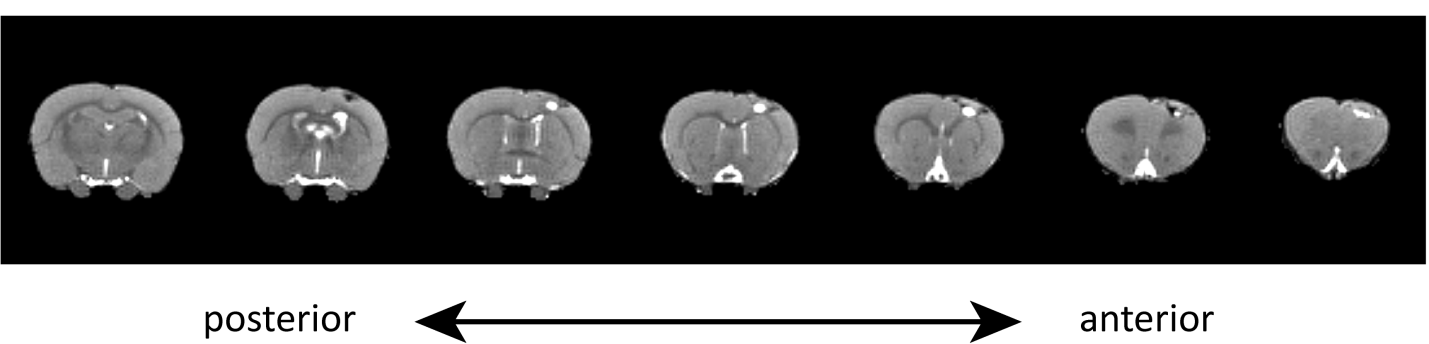


***Figure 1: MRI of stroke lesion.*** *Anatomical MR images of consecutive coronal rat brain slices at 17 days after photothrombotic stroke, revealing a unilateral lesion in the sensorimotor cortex. MRI data showed no signs of damage to the subcortical striatum. Signal changes were occasionally observed in white matter underlying the lesion, which may be attributed to microhemorrhages.*

*Skilled reaching*

Skilled reaching success rate, expressed as the percentage of successfully obtained pellets out of 2-20 reaching trials, dropped from 38 ± 17% before stroke to 17 ± 16% at 3 days after stroke in rats (**Figure 2**). Partial recovery to 23 ± 20% was observed after 23 days.

***Figure 2: Pellet reaching success rate at different time-points before and after stroke in rats.*** *Data are presented as means ± standard deviation.*
